# Lacking catalase, a protistan parasite draws on its photosynthetic ancestry to complete an antioxidant repertoire with ascorbate peroxidase

**DOI:** 10.1186/s12862-019-1465-5

**Published:** 2019-07-19

**Authors:** Eric J. Schott, Santiago Di Lella, Tsvetan R. Bachvaroff, L. Mario Amzel, Gerardo R. Vasta

**Affiliations:** 1 Department of Microbiology & Immunology, University of Maryland School of Medicine, and Institute of Marine and Environmental Technology, 701 E. Pratt Street, Baltimore, MD 21202 USA; 2 Instituto de Química Biológica - Ciencias Exactas y Naturales, IQUIBICEN / CONICET, Departamento de Química Biológica, Fac. de Cs. Exactas y Naturales, Universidad de Buenos Aires, Ciudad Universitaria, Capital Federal, Argentina; 3 University of Maryland Center for Environmental Science, Institute of Marine and Environmental Technology, 701 E. Pratt Street, Baltimore, MD 21202 USA; 4 Department of Biophysics & Biophysical Chemistry, The Johns Hopkins University School of Medicine, 725 North Wolfe Street, Baltimore, MD 21205 USA; 5 Present address: University of Maryland Center for Environmental Science, Institute of Marine and Environmental Technology, 701 E. Pratt Street, Baltimore, MD 21202 USA

**Keywords:** Ascorbic acid, Ascorbate peroxidase, Hydrogen peroxide, Oxidative stress, Parasite, *Perkinsus marinus*, Vacuole

## Abstract

**Background:**

Antioxidative enzymes contribute to a parasite’s ability to counteract the host’s intracellular killing mechanisms. The facultative intracellular oyster parasite, *Perkinsus marinus*, a sister taxon to dinoflagellates and apicomplexans, is responsible for mortalities of oysters along the Atlantic coast of North America. Parasite trophozoites enter molluscan hemocytes by subverting the phagocytic response while inhibiting the typical respiratory burst. Because *P. marinus* lacks catalase, the mechanism(s) by which the parasite evade the toxic effects of hydrogen peroxide had remained unclear. We previously found that *P. marinus* displays an ascorbate-dependent peroxidase (APX) activity typical of photosynthetic eukaryotes. Like other alveolates, the evolutionary history of *P. marinus* includes multiple endosymbiotic events. The discovery of APX in *P. marinus* raised the questions: From which ancestral lineage is this APX derived, and what role does it play in the parasite’s life history?

**Results:**

Purification of *P. marinus* cytosolic APX activity identified a 32 kDa protein. Amplification of parasite cDNA with oligonucleotides corresponding to peptides of the purified protein revealed two putative APX-encoding genes, designated *PmAPX1* and *PmAPX2*. The predicted proteins are 93% identical, and PmAPX2 carries a 30 amino acid N-terminal extension relative to PmAPX1. The *P. marinus* APX proteins are similar to predicted APX proteins of dinoflagellates, and they more closely resemble chloroplastic than cytosolic APX enzymes of plants. Immunofluorescence for PmAPX1 and PmAPX2 shows that PmAPX1 is cytoplasmic, while PmAPX2 is localized to the periphery of the central vacuole. Three-dimensional modeling of the predicted proteins shows pronounced differences in surface charge of PmAPX1 and PmAPX2 in the vicinity of the aperture that provides access to the heme and active site.

**Conclusions:**

PmAPX1 and PmAPX2 phylogenetic analysis suggests that they are derived from a plant ancestor. Plant ancestry is further supported by the presence of ascorbate synthesis genes in the *P. marinus* genome that are similar to those in plants. The localizations and 3D structures of the two APX isoforms suggest that APX fulfills multiple functions in *P. marinus* within two compartments. The possible role of APX in free-living and parasitic stages of the life history of *P. marinus* is discussed.

**Electronic supplementary material:**

The online version of this article (10.1186/s12862-019-1465-5) contains supplementary material, which is available to authorized users.

## Background

*Perkinsus marinus* is a facultative intracellular protistan parasite of the eastern oyster (*Crassostrea virginica*) responsible for economically and environmentally devastating declines in oyster populations from the Gulf of Mexico to New England [1, 2]. Other species within the genus are associated with mortalities of important shellfish species worldwide [3]. Initially described as a fungus (*Dermocystidium marinum*), subsequently as an apicomplexan parasite due to the presence of an apical complex in the flagellated stage, and finally as a dinoflagellate, *P. marinus* remained as an *incertae sedis* until the phylum Perkinsozoa was established to include all *Perkinsus* spp. [4, 5]. Phylogenetic analyses based on single or multiple genes have placed the phylum Perkinsozoa in a key position between the apicomplexans, which are obligate parasites, and dinozoans, which are mostly photosynthetic free living and parasitic species [6–8]. The life cycle of *Perkinsus* spp. includes both a flagellated free living (zoospore) stage and an intracellular stage (trophozoite) that proliferates within the hemocytes of host mollusks [3, 4].

Perkinsozoa, Dinozoa and Apicomplexa share a common ancestor that may have been a photosynthetic eukaryote. Vestiges of this photosynthetic ancestry persist in structures and biochemical pathways derived from a presumed photosynthetic endosymbiont [6–8]. Unlike some Apicomplexans and all dinoflagellates, *Perkinsus* spp. do not possess an obvious plastid or relict plastid. They do, however, contain a prominent vacuole that develops within both trophozoites and zoospores [4, 9]. Within the vacuole are electron dense bodies known as vacuoplasts, which have been well-described structurally, but whose function remains unknown [4, 10], though they have been hypothesized to represent an energy storage reserve [11]. Further, it remains unclear whether there are evolutionary or functional relationships between the *Perkinsus* vacuole and vacuoles of related protists such as *Plasmodium falciparum*, which is the site of hemoglobin degradation, dinoflagellates, which serves to digest engulfed prey, or green algae, in which the vacuole has a structural function of increasing cell size and rigidity [12–15].

*P. marinus* trophozoites enter molluscan hemocytes by subverting the phagocytic response [16–18] while abrogating the typical respiratory burst by suppressing the accumulation of reactive oxygen species (ROS) [19–21]. Thus, the intracellular survival and proliferation of the parasite is enabled by a complex and effective antioxidative enzyme repertoire [22–25]. Oyster hemocytes generate superoxide (OH^.^), hydrogen peroxide (H_2_O_2_), and hypochlorous acid (HOCl) [20, 26, 27]. Like apicomplexans and dinoflagellates, perkinsozoans possess multiple Fe-cofactored SODs, but lack Cn/Zn and Mn-type SODs. In *P. marinus,* one of the two FeSODs (PmSOD1) is targeted to the mitochondrion, while the other (PmSOD2) is distributed to punctate regions at the margin of the cell under the plasma membrane [23]. Mitochondrially-targeted FeSOD isoforms are also described for the apicomplexan parasites (*Toxoplasma* and *Plasmodium*) and dinoflagellates (*Symbiodinium*) [28–30]. FeSODs of *P. marinus* are capable of removing superoxide, [23–25], but the product of SOD activity is yet another ROS, H_2_O_2_. The uncharged hydrogen peroxide is capable of passing through cell membranes, and is the substrate for host myeloperoxidase (MPO). MPO activity on H_2_O_2_ produces HOCl [27], which in the presence of iron produces the highly reactive hydroxyl radical (^.^OH) via the Fenton reaction [31]. Most organisms, with the exception of a few protists and unicellular algae, contain catalase (EC 1.11.1.6) that readily converts H_2_O_2_ to water and molecular oxygen (2 H_2_O_2_ → 2 H_2_O + O_2_), and prevents the formation of the highly cytotoxic HOCl and ^.^OH [32, 33]. In all kingdoms of life, H_2_O_2_ removal is also carried out by diverse heme peroxidases (generally H_2_O_2_ + 2AH_2_ → H_2_O + 2AH), which use an array of electron acceptors [34], and thiol-dependent peroxidases such as peroxiredoxins and glutathione peroxidase [35].

Transcriptomic and genomic databases of *Perkinsus* spp. appear to lack catalase genes [36, 37]. Additionally, cytosolic *P. marinus* extracts lack catalase activity and instead destroy H_2_O_2_ by an ascorbate dependent peroxidase activity (APX, EC 1.11.1.11) [22], which is typically found in photosynthetic eukaryotes. Herein, we describe the purification of *P. marinus* APX activity and the characterization of two genes encoding highly similar APX proteins that have different N-terminal leaders and several short peptide motifs that differ between the two. A phylogenetic analysis of the *P. marinus* APX proteins was conducted relative to APX proteins from related protists and higher plants. Isoform-specific antibodies were used to visualize the subcellular locations of the two APX isoforms and computer-assisted 3D modeling was performed to predict the consequences of the divergent sequence motifs on heme and substrate access within the APX structures. The localization, predicted structures and phylogenetic analyses are discussed in the context of the facultative intracellular parasitic life history of *P. marinus*.

## Results

### Purification of *P. marinus* ascorbate peroxidase activity

**Fig. 1:**
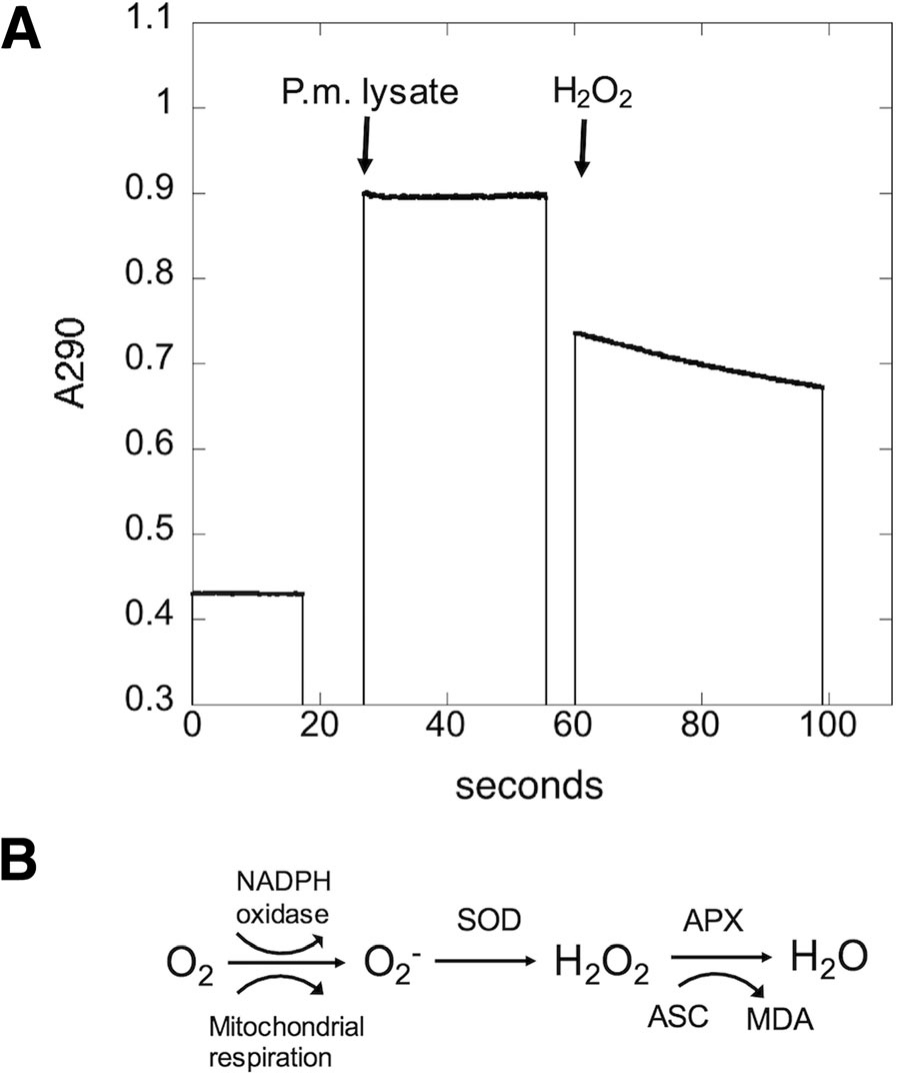
Kinetics trace of hydrogen peroxide-dependent destruction of ascorbate by *P. marinus* lysate. **a** *P. marinus* osmotic lysate was added to assay buffer at 26 s. At 60 s, 0.1 mM hydrogen peroxide was added and ascorbate content was monitored at 290 nm. Both assay buffer and *P. marinus* lysate contained 0.4 mM ascorbate. **b** Proposed pathway for production and destruction of hydrogen peroxide in *P. marinus*, based on the presence of APX and lack of catalase activity

**Fig. 2:**
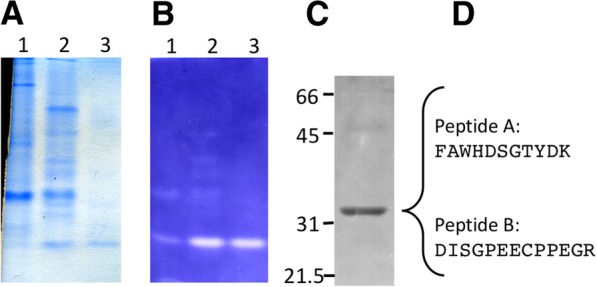
PAGE analysis of APX activity and protein during the purification of APX activity. Peak APX fractions were separated by nondenaturing 10% PAGE. **a** Gel stained with Coomassie blue. **b** APX activity was visualized by a nitroblue tetrazolium stain. Lanes 1: Peak APX fraction from DEAE sephacel (30 μg protein). Lanes 2: Peak APX fraction from phenyl Sepharose, Lanes 3: The fraction following the APX peak activity from phenyl Sepharose. **c** SDS-PAGE analysis of peak APX fraction. **d** Sequences of tryptic peptides identified by Edman degradation from the 32 kDa band in (**c**)

Osmotic lysates of *P. marinus* trophozoites possess APX activity, detectable as the H_2_O_2_-dependent oxidation of ascorbate (Fig. 1). Beginning with an osmotic lysate that had a specific APX activity of 3.8 mM min^− 1^ mg^− 1^ protein, APX activity was purified first through ion exchange (DEAE-Sephacel), followed by hydrophobic interaction (phenyl Sepharose) chromatography to enrich the activity 1170-fold with a 40% yield (Table 1). In the crude extract and DEAE-Sepharose stages, the APX activity was unstable without ascorbate in the buffer, similar to plant APX activities [38]. Osmotic extracts displayed no detectable H_2_O_2_-dependent oxidation of cytochrome c (data not shown). When diluted in buffer with 1.5 M (NH_4_)_2_SO_4_, APX activity remained stable without ascorbate in the buffer (Experimental Procedures and data not shown). The fractions with peak APX activity, when separated by 10% nondenaturing PAGE and analyzed by an in-gel APX assay [39], displayed APX activity associated with a single dominant Coomassie-staining band (Fig. 2a,b). SDS-PAGE analysis of these peak fractions showed a major protein of 32 kDa (Fig. 2c). To obtain additional protein for sequence analysis, the purification procedure was repeated, with an additional stage of MonoQ-Sepharose chromatography included after the DEAE-Sephacel stage. The 32 kDa band from this purification was subjected to in-gel tryptic digestion, HPLC separation, and peptide analysis by MS/MS and Edman degradation. Two peptide sequences were obtained: Peptide A: FAWHDSGTYDK and Peptide B: DISGPEECPPEGR (Fig. 2d). BLAST analysis identified these peptides as similar to APX enzymes from chloroplasts of plants (data not shown).

**Table 1:** Ascorbate peroxidase purification from *P. marinus*

Enrichment Step	Total protein *mg*	Specific activity *μM min*^*− 1*^ *mg*^*− 1*^	Yield *%*	Purification *-fold*
Osmotic lysis	16.1	0.49	100	1.0
DEAE sephacel	2.85	6.63	238^a^	13.7
Phenyl sepharose	0.033	493.5	206^a^	1007

^a^Yield of > 100% may reflect the removal of an inhibitor in the DEAE step

### Identification and characterization of two ascorbate peroxidase genes

**Fig. 3:**
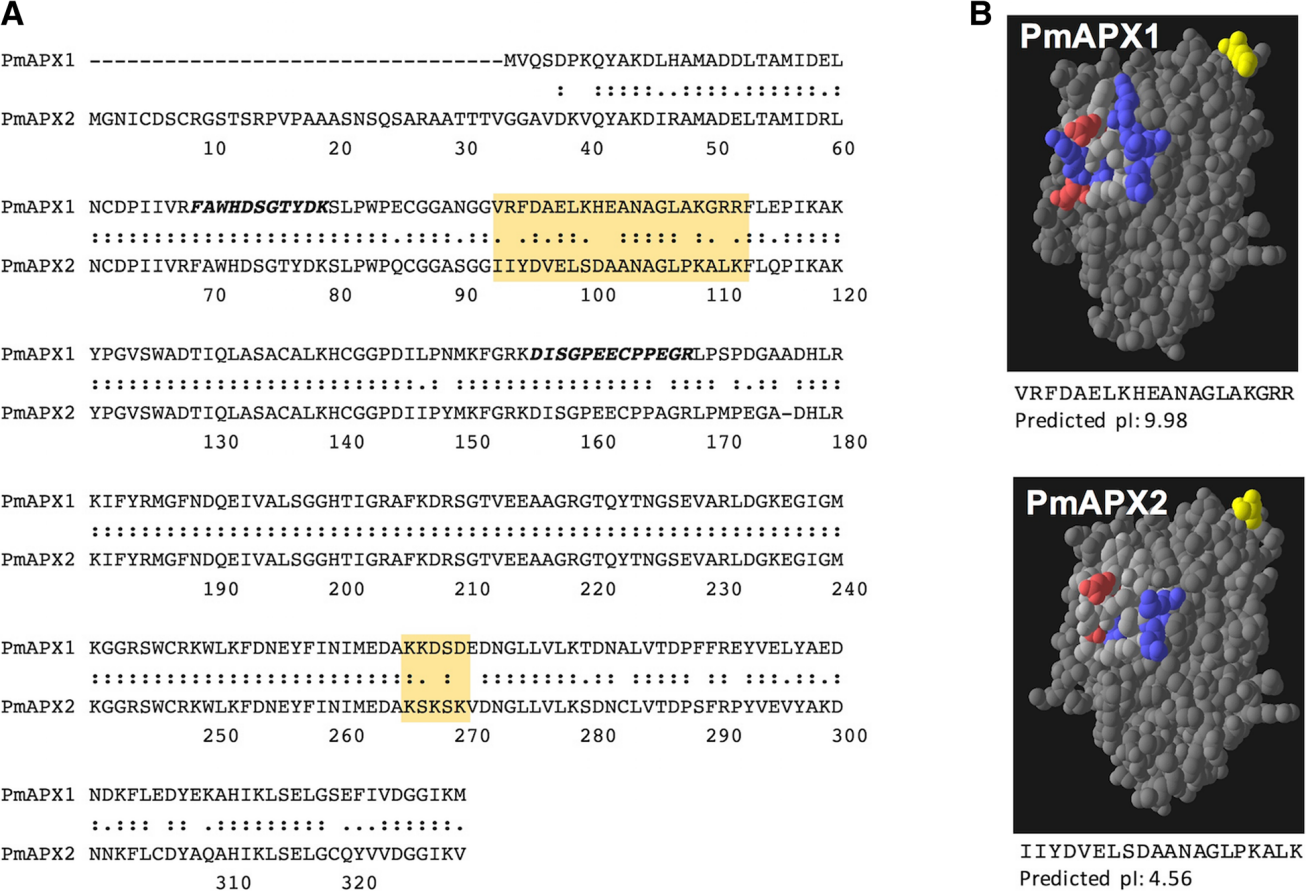
Comparison of the two predicted *P. marinus* APX proteins. **a** Alignment of PmAPX1 and PmAPX2 amino acid sequences. Amino acid numbering refers to PmAPX2. The shaded areas highlight regions that differ most between the two proteins. Peptides identified by MALDI are in bold italics, the peptide at position 156–168 corresponds to Peptide B in Fig. 2d. **b** 3-dimensional models of PmAPX1 and PmAPX2 based on the crystal structure of soybean APX. Residues are color coded to highlight the region identified in part A. Acidic residues are red while basic residues are blue. The N-terminal methionine of each protein is shown in yellow

Degenerate oligonucleotide primers were designed, based on sequences of Peptides A and B, and used to PCR amplify a 261 bp product from a *P. marinus* cDNA library. The sequence of this amplicon was similar to published APX gene sequences (not shown), and was used to design specific primers to amplify towards the 5′ and 3′ ends of the putative APX gene, in conjunction with primers in the cDNA library cloning vector (lambda Zap). The 5′ and 3′ end amplifications resulted in the identification of two distinct APX genes, which were designated *PmAPX1* and *PmAPX2* (identical to sequences in GenBank accession numbers XM_002767244 and XM_002767239, respectively). *PmAPX1* encodes a 297 amino acid protein predicted to be 32,981 Da, with a pI of 5.1. The product of *PmAPX2* is predicted to be 329 amino acids with a mass of 35,676 Da, and pI of 7.9. The precise sequence of the experimentally-determined Peptide A is found in both predicted proteins (residues 69–80 in Fig. 3a). Peptide B, however, is an exact match only for PmAPX1, and has a single substitution (Glu165➔ Ala165) relative to PmAPX2 (see residues 156–168 in Fig. 3a). The *PmAPX1* and *PmAPX2* genes did not appear to be tightly linked, based on blots of restriction enzyme-digested DNA probed separately with ^32^P-labeled PCR products corresponding to the 5′ untranslated regions of *PmAPX1* and *PmAPX2* (see Additional file 1: Figure S1). Inspection of *P. marinus* genome scaffold contig NW_003202288 shows that the two genes are approximately 12 kb apart.

### Preliminary analyses of PmAPX1 and PmAPX2 structures

The amino acid sequences of PmAPX1 and PmAPX2 are 84% identical and 93% similar over 292 amino acids, excluding the 32 amino acid N-terminal extension in PmAPX2. The predicted leader on PmAPX2 lacks any obvious transmembrane motif and has a pI of 9.01. In addition to the leader peptide, the most notable difference between PmAPX1 and PmAPX2 is a 20 amino acid-long region (residues 92–112 in Fig. 3a), that is basic (pI of 9.98) in PmAPX1 and acidic (pI of 4.56) in PmAPX2. To explore the charge and topology of this 20 residue long region we developed three-dimensional predictions, using as a template the crystal structure of the chloroplastic APX of tobacco (PDB ID 1iyn; [40]). The models (Fig. 3b, created in Swiss PDBViewer 3.0) indicated that this region is in a solvent-exposed loop of the tobacco APX. The different amino acid sequences and charges of this surface-exposed region provided guidance for raising PmAPX1 and PmAPX2 isoform-specific antibodies, which were used to detect each gene product.

### Phylogenetic relationships of PmAPX1 and PmAPX2

We investigated the structural and phylogenetic relationships of PmAPX1 and PmAPX2 to APXs identified in related protists and photosynthetic organisms. The initial search for putative APX from core dinoflagellates, using *P. marinus* sequences as queries, revealed multiple high scoring hits present in each dinoflagellate species’ transcriptome dataset (available at TreeBase.org as study 23,146, and listed in Additional file 2: Table S1). These hits could be roughly binned into two categories based on identity of 45% and e-values <1e-50 for one category and pairwise identity of 35% with e-values of 1e-40 to 1e-20 for the second. For example, BLAST comparison of the *Karlodinium veneficum* with PmAPX1 identified one protein (DQ118600.1, ABA55544.1) with 48% identity (e value 1e-90), and another (ADV9123.1) with 30% identity (e value 2e-37) to PmAPX1. In the Peroxibase scheme of characterizing type I heme peroxidases [41], the former protein is a “monofunctional ascorbate peroxidase” (APx) [42], whereas the latter *K. veneficum* protein is in the hybrid “APX-cytochrome c peroxidase” category (APx-CcP). BLAST comparison to *Vitrella brassicaformis* identified a single protein (e value 4e-82) categorized as a typical APX-like monofunctional ascorbate peroxidase. In contrast, BLAST of PmAPX1 to the kinetoplastid *T. cruzi*, which possess only hybrid APx-CcP-type peroxidases, shows matches lower than 35% identity and e values above 4e-35.

**Fig. 4:**
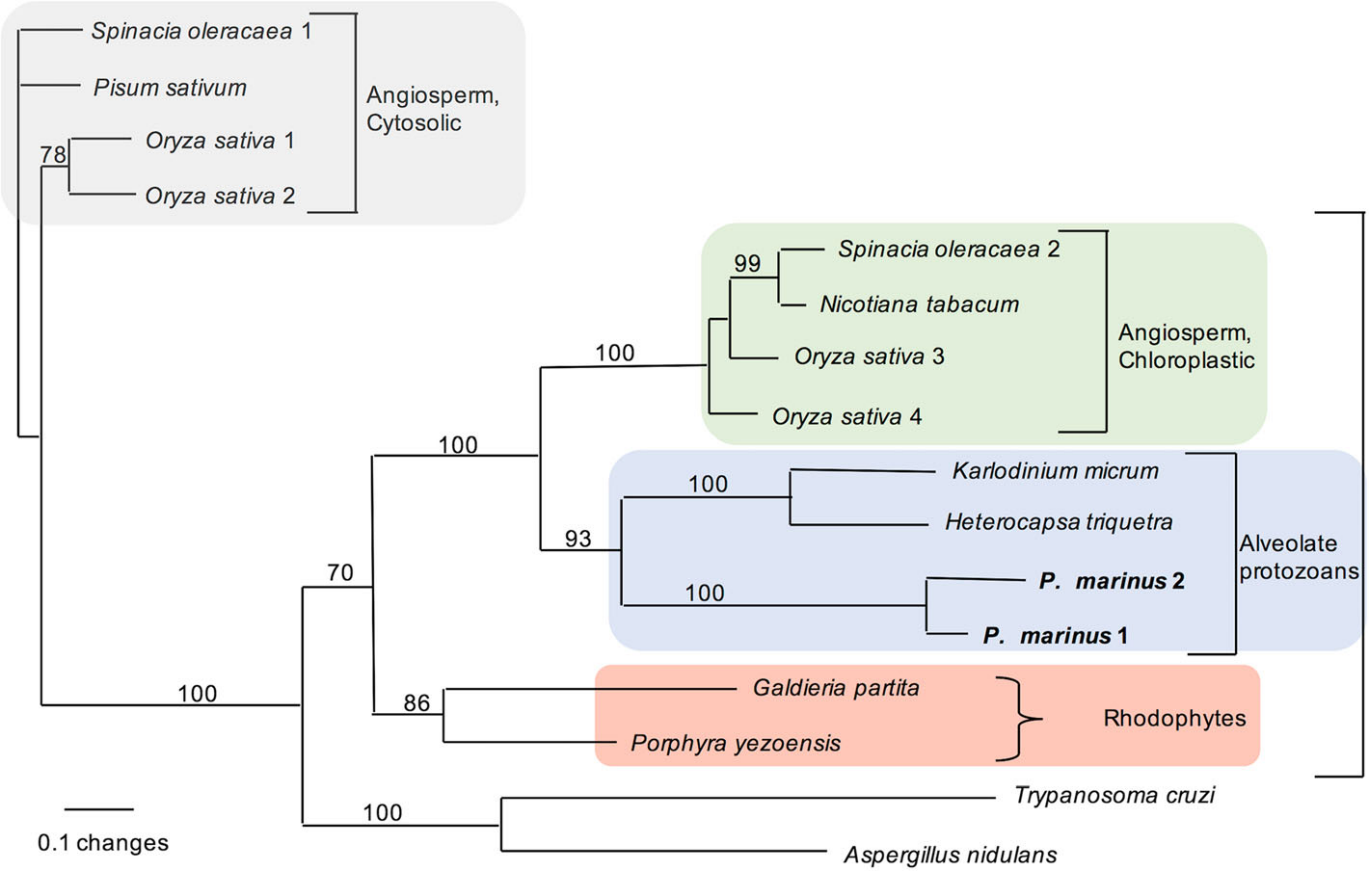
Phylogenetic relationship of PmAPX1 and PmAPX2 to predicted APX proteins of other photosynthetic taxa. Maximum likelihood relationships of PmAPX1 and PmAPX2 in relation to APX from plants and protozoans. Outgroups are the APX-like proteins of *T. cruzi* and *A. nidulans. P. marinus* lies firmly within the plastidic branch of tree, which is deeper than the branch between rhodophytes, alveolates and plastids. Bootstrap values are based on 100 replicates. Square brackets indicate monophyletic lineages. Curly brackets identify taxonomic groupings. Genbank numbers for proteins are as follows: *Spinacia oleracaea* 1, BAA12890*; Spinacia oleracaea* 2, BAA19611*; Nicotiana tabacum,* BAC10691*; Oryza sativa* 1, A2XFC7*; Oryza sativa 2, Q9FE01; Oryza sativa* 3, BAC79363*; Oryza sativa* 4*,* BAC79362*; Pisum sativum,* 1APX*_A; Pyropia yezoensis,* BAD16708*; Galdieria partita,* BAC41199*; Trypanosoma cruzi,* CAD30023*; Aspergillus nidulans,* XP_663044*; Karlodinium micrum,* DQ118600*; Heterocapsa triquetra,* AY826833

**Fig. 5:**
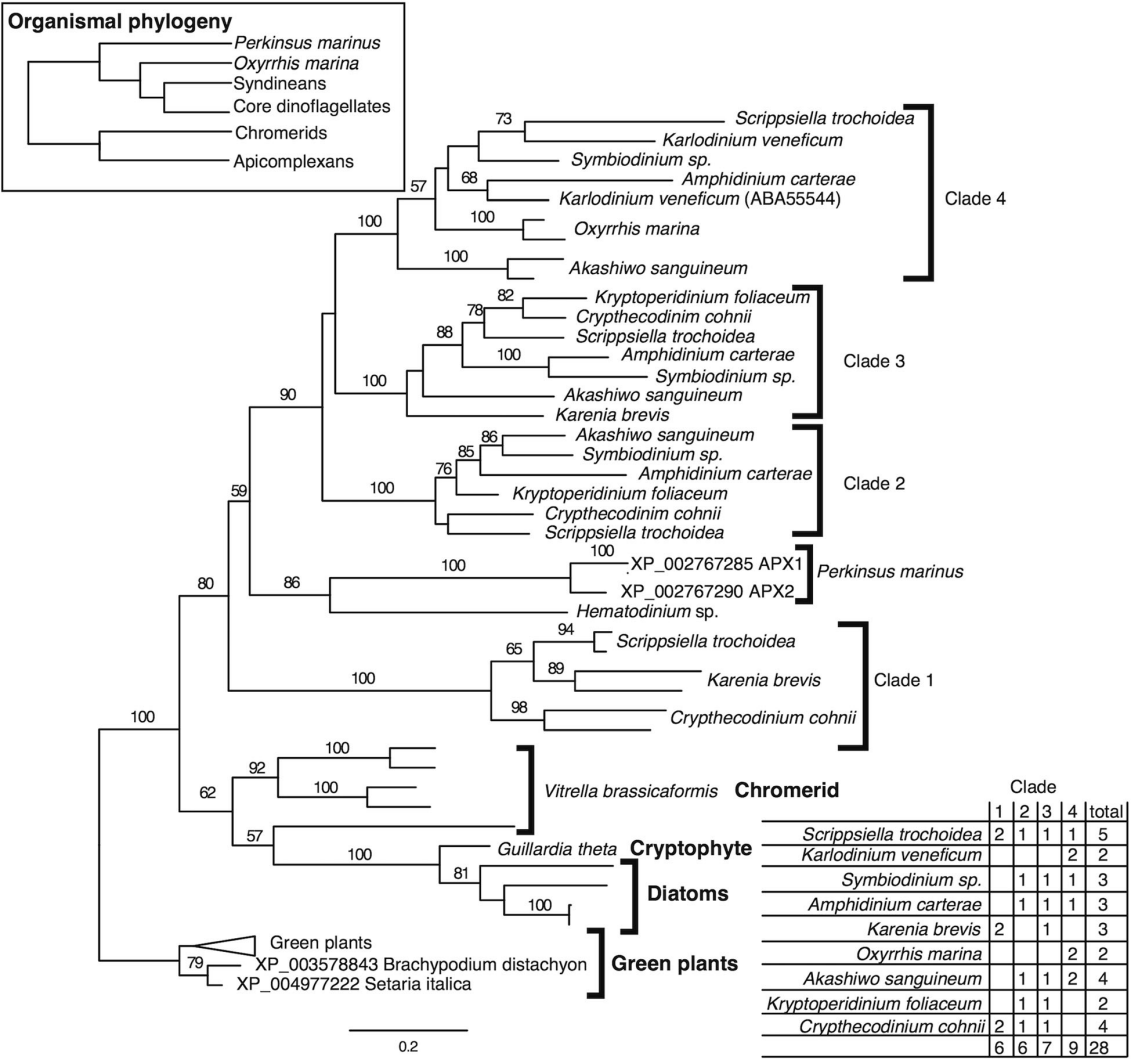
Phylogeny of predicted APX proteins within the perkinsids and dinoflagellates. *P. marinus* APXs are distinct from all four dinoflagellate clades, and share a weakly supported group with *Hematodinium* sp. The tree shows representative core dinoflagellates that possess from one to four of the clades. The basal dinoflagellate *Oxyrrhis* contains only clade 4 APX form (inset table lower right). Organismal taxonomic relationships are drawn in the inset, upper left

Starting with *A. carterae*, the high scoring matches were collected and appended to the *P. marinus* queries. The BLAST results suggested at least three or more different high scoring matches were present in each core dinoflagellate transcriptome. Phylogenetic analysis with a broad range of outgroups and both cytosolic and chloroplast versions of APX from plants was congruent with a plastid-type APX in *P. marinus*. This was confirmed by BLAST queries using a full range of plant APX protein sequences, suggesting the initial search had successfully extracted the full diversity of APX in dinoflagellates, chromerids and the other chlorophyll c pigmented algae. No BLAST hits for APX in apicomplexans were found when using taxon-specific databases at either the level of Apicomplexa as a whole or at the genus level. The initial phylogeny showed a green plastid-type clade of APX were present in *P. marinus* and dinoflagellates and a second clade included putative cytosolic APX from core dinoflagellates, diatoms and trypanosomes, and red algae (Fig. 4). The cytosolic outgroup was removed in an attempt to further refine the relationships between types of APX in dinoflagellates, chromerids and chlorophyll c algae. The initial BLAST-based census against the *P. marinus* transcriptome suggested *P. marinus* expresses only the two APX isoforms, while most core dinoflagellates express between 2 and 5 APX genes. In addition, there were multiple green plastid-type APX in diatoms and the chromerid *Vitrella brassicaformis*. The more focused phylogeny (Fig. 5), rooted on the green plant chloroplast APX (100% bootstrap support), showed a poorly supported clade (62%) that encompasses diatoms, cryptophyte and chromerid sequences forming an outgroup to the moderately supported clade (80% bootstrap) including 4 distinct dinoflagellate clades, *Hematodinium* sp., *Oxyrrhis* and *P. marinus*. The overall phylogeny is not congruent with organismal relationships (see inset in Fig. 5), which place *Oxyrrhis* and *P. marinus* as outgroups to *Hematodinium* and the core dinoflagellates [43]. However, the APX phylogeny suggests the different core dinoflagellate clades and the two APX from *P. marinus* share a common origin, with independent duplication and likely loss of genes from some lineages. The phylogeny suggests that duplication of APX in *P. marinus* and *V. brassicaformis* occurred relatively recently in comparison to multiple shared duplications within core dinoflagellates. *Perkinsus marinus* APX does not have a clear affinity with any of the four core dinoflagellate APX clades. The two *Oxyrrhis* sequences available fall within clade 1 of the dinoflagellate group. Across core dinoflagellates there may be either differential loss or incomplete sampling, as not all clades/isoforms have representatives in every one of the core dinoflagellate species. Indeed clade 1 contains two distinct versions but only three species, while clade 3 is the most complete with representatives from seven species. From a single species perspective, *Kryptoperidinium foliaceum* contained only two detectable versions, while *Scrippsiella trochoidea* contained five, with at least one sequence from each major clade.

### Subcellular localization of PmAPX1 and PmAPX2

**Fig. 6:**
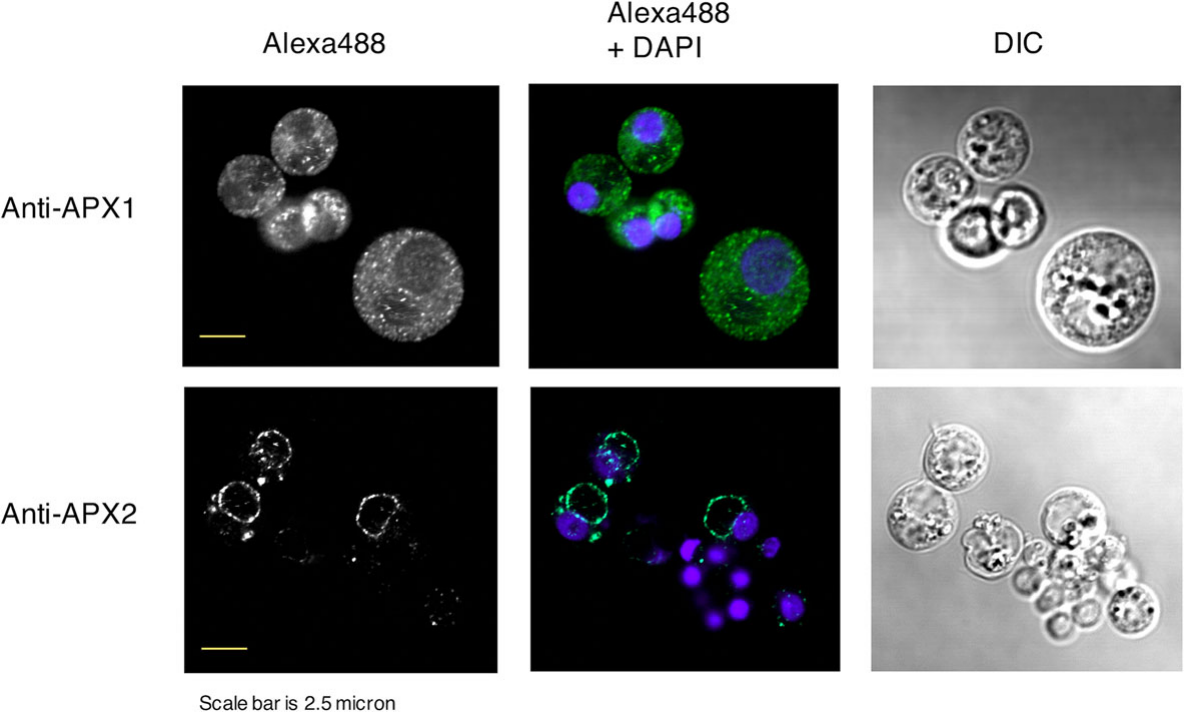
Indirect immunofluorescence of PmAPX1 and PmAPX2 in cultured *P. marinus* trophozoites. Early log phase cultures were fixed, stained with PmAPX1- or PmAPX2-specific IgG, followed by Alexa488 labeled secondary antibody. Nuclei were visualized with DAPI. PmAPX1 localized to the cytoplasm and PmAPX2 localized to the margin the vacuoles. Bar = 2.5 μm. DIC = differential interference contrast imaging

To visualize the cellular locations of PmAPX1 and PmAPX2 in *P. marinus*, isoform-specific antibodies were raised against synthetic peptides corresponding to the region (amino acids 93–113, Fig. 3) that differs between PmAPX1 and PmAPX2. Protein blots of cultured *P. marinus* trophozoites showed that anti-PmAPX1 and anti-PmAPX2 antisera identified single bands of approximately 32 and 35 kDa, respectively (see Materials and Methods and Additional file 3: Figure S2). Immunofluorescence of-PmAPX1 and anti-PmAPX2 with isoform-specific antisera in fixed, permeablized *P. marinus* trophozoites (Fig. 6) showed PmAPX1 labeling throughout the cytoplasm of large and small trophozoites. The plasma membrane and vacuolar membrane were not specifically stained, nor was the nuclear margin stained. In contrast, anti-PmAPX2 serum labeled the vacuolar membrane, but not the plasma membrane, nuclear membrane or cytoplasm. In some cells, tiny punctate labeling was visible in cytoplasm, and in others there were bright condensed spots seen adjacent to the nucleus. No labeling with anti-PmAPX2 was seen in small trophozoites that had not yet developed a central vacuole.

### Homology modeling of PmAPX1 and PmAPX2 structures

**Fig. 7:**
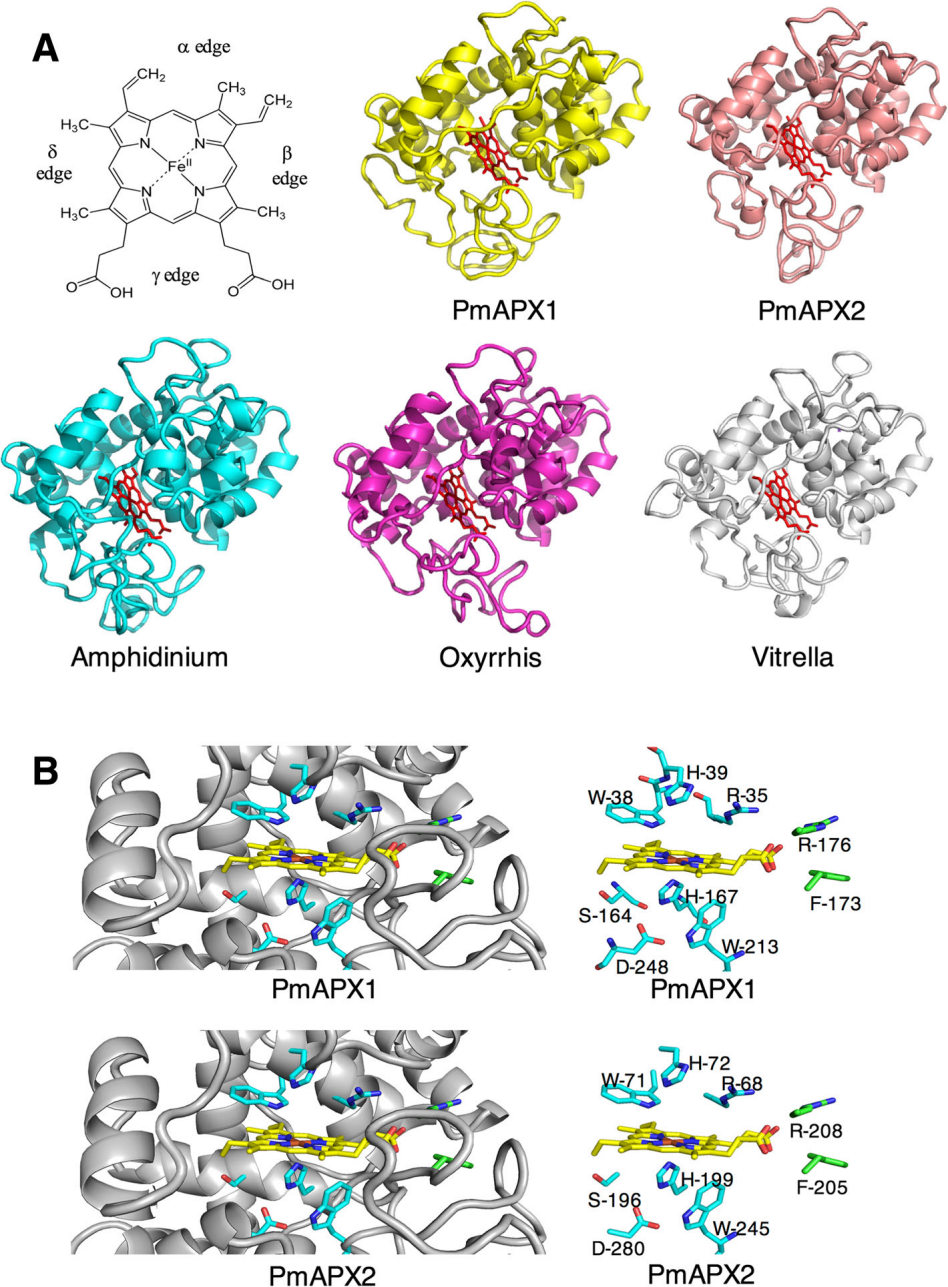
Homology models of PmAPX1, PmAPX2 and predicted APX proteins from *Oxyrrhis*, *Amphidinium*, and *Vitrella.* **a** Ribbon diagrams, illustrating a high degree of conserved alpha helix structure above the heme plane and along the plane edges. Below the heme plane the models diverge and there is less helical structure. Apertures to the heme group through equatorial gaps in the modeled surface are visible in this view. The top left structure is a heme molecule diagram with alpha, beta, delta and gamma edges labeled. **b** The active site of PmAPX1 and PmAPX2, showing conserved structure and residues. Left: Ribbon diagrams showing the residues in the vicinity of the heme (Carbon: yellow; Nitrogen: blue; Oxygen: red). Right: The heme group and nearby residues are shown without the peptide backbone. The conserved residues Trp-His-Arg-Asp above the heme and Ser-His-Trp-Asp below the heme are labeled (Carbon: light blue; Nitrogen: blue; Oxygen: red). The Phe residue (173 in APX1, 205 in APX2) (Carbon: green; Nitrogen: blue; Oxygen: red) at the gamma heme edge is conserved between PmAPX1, PmAPX2 and the APX of dinoflagellates, but not in plants or *Vitrella* (where the corresponding residue is His or Arg, see Additional file 4: Figure S3)

Using as a template the X-ray structure of tobacco stromal APX (PDB ID 1iyn), models were produced for the two *Perkinsus* APX as well as APX from *Oxyrrhis, Amphidinium,* and *Vitrella*. All 5 structures have the organization shared by this class of heme peroxidases [44]. There are 13 alpha helices arranged in two spatial domains that enclose the active site heme and substrate cavity (Fig. 7). Seven helices are arranged above the plane of the heme and on the beta heme edge, and 6 helices wrap from below the heme around the alpha heme edge (Ser 160, Trp and Arg) [40].

In both PmAPX1 and PmAPX2 the helices closest to the heme group hold in place a number of well-conserved residues that interact, either directly or through water molecules, with the heme and ascorbate substrate in other APX proteins [45, 46]. A His residue (position 39 in PmAPX1, 72 in PmAPX2) resides directly above the heme iron atom. This is flanked by a Trp residue (position 38 in PmAPX1, 71 in PmAPX2) and Arg residue (position 35 in PmAPX1, 68 in PmAPX2) that comprise the catalytic triad that together transfer oxygen from H_2_O_2_ to the heme molecule to create compound I [heme Fe4+ radical] [40]. A second Arg residue (176 in PmAPX1, 208 in PmAPX2) lies closer to the plane of the heme, near the propionate moiety of the gamma heme edge. Also at the gamma heme edge is a conserved Phe residue that is present in both of the *P. marinus* APXs and in APX from dinoflagellates, but not present in plants or the Chromerid, *Vitrella* (Fig. 7). This Arg is known to rotate into position to contact ascorbate [46]. Below the plane of the heme (Fig. 7), another His (167 in PmAPX1, 199 in PmAPX2) lies directly beneath the iron atom, and is flanked by a trio of conserved residues: Ser, Asp, and Trp that in crystal structure analyses are shown to hold the His in its position [45].

**Fig. 8:**
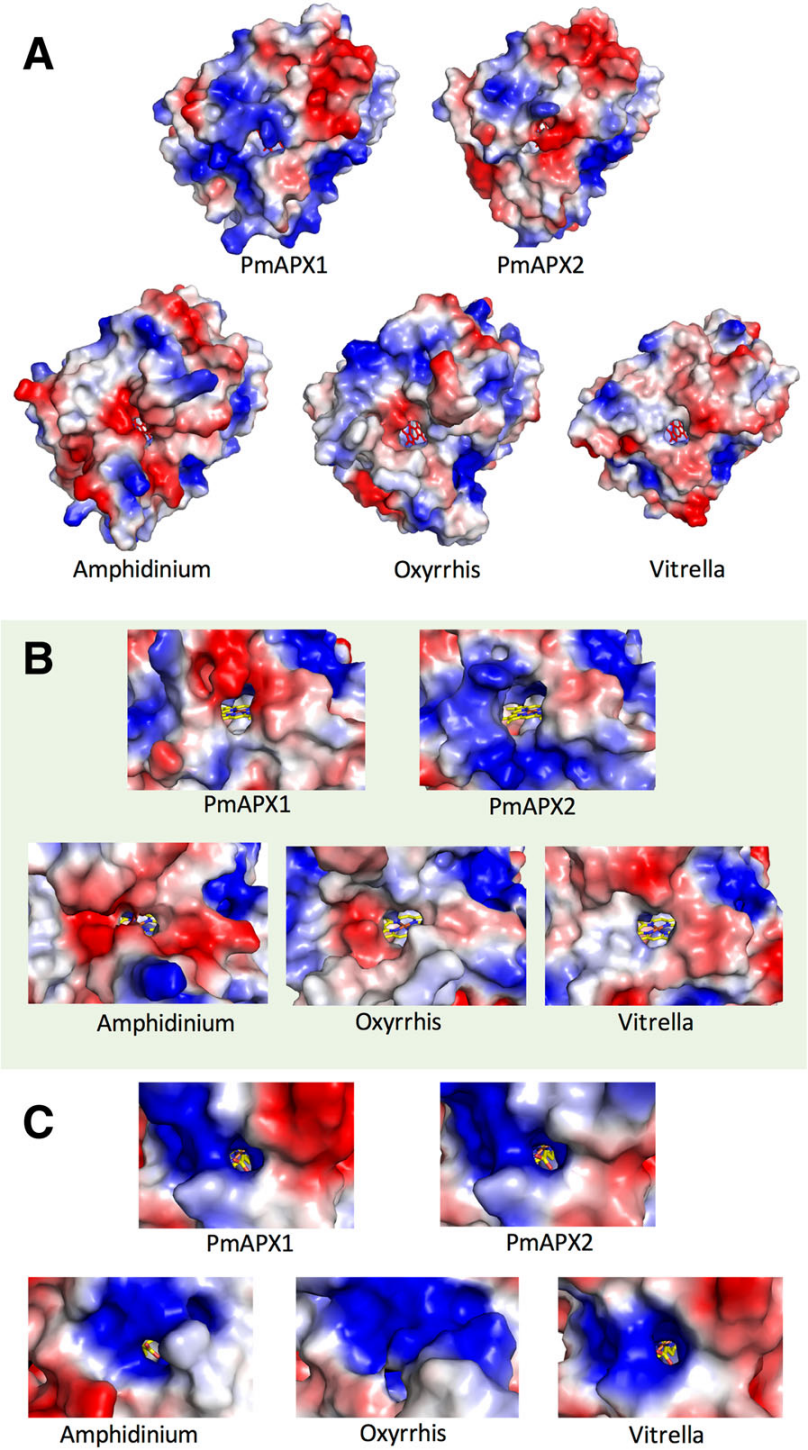
Surface charge comparisons of PmAPX1 and PmAPX2. **a** Electrostatic potential surface based on the surface charges of the face presented in Fig. 7a. Negatively charged (acidic) surfaces in red, positive (basic) charges in blue and neutral charged surfaces in white. **b** Surface charge representation at the aperture leading to the delta heme edge. **c** Surface charge representation at the aperture leading to the gamma heme edge

Non-helical structure predominates below the heme plane and on the delta and gamma heme edges (Fig. 7a). The non-helical domains also comprise the two sides of the protein that form the apertures to the active site within (Fig. 8). Both apertures are oriented roughly in the plane of the heme, with the wider aperture (8.5 Å; 12.0 Å main-chain to main-chain) to the delta heme edge and the narrower aperture (3–4 Å) facing the gamma heme edge, and towards which the propionate moieties project (it is in this region that the ascorbate binds [45]). The charge of the PmAPX1 delta heme edge aperture and surrounding surface is negative to neutral (red and white in Fig. 8), and similar to that of *Amphidinium, Oxyrrhis* and *Vitrella*. In contrast, the PmAPX2 delta heme edge aperture and surrounding surface is highly positive (blue in Fig. 8b), and presents a slightly wider opening than PmAPX1 or the other three species. The channel of the gamma heme edge aperture of all five proteins, including PmAPX1 and PmAPX2, carries a positive charge (Fig. 8c), and is oriented towards the propionate structure of the heme molecule within. The entrance to the gamma channel of the *Oxyrrhis* APX model is occluded by the side chains of two large residues, Arg 39 and Arg 197 (blue area in the *Oxyrrhis* model, Fig. 8b). Nevertheless, there is still an entrance for the substrate slightly below those of the other proteins, opened by the small side chain of Ala 193.

The sequences of PmAPX1 and PmAPX2 both contain a 6-residue insertion that is not present in the APX of any of the other related species (see alignment Additional file 4: Figure S3). These residues reside in the non-helical portion well below the plane of heme. The orientation of this loop differs between PmAPX1 and PmAPX2, as illustrated in a backbone overlay of the two predicted molecules. Both loops carry charged side chains, making them highly interactive with solvent. The motif in PmAPX1 (AKKDSD, residues 264–269) has two negative aspartate residues that charge balance the positive lysines (see Additional file 5: Figure S4). The loop of PmAPX2 (AKSKSK) has an overall positive charge, driving it into the solvent phase and away from the main mass of the protein. The carboxyl end of the loop in both PmAPX1 and PmAPX2 is anchored by a stretch of hydrophobic residues (GLLVL).

## Discussion

The removal of H_2_O_2_ is essential for aerobic life, and an enhanced H_2_O_2_ removal system is a crucial function in parasites to counter the host innate immune ROS cascade [47]. Prior work showed that the oyster parasite *P. marinus* suppresses ROS accumulation in its host, and displays resistance to H_2_O_2_, yet had no evidence of catalase activity (25). Furthermore, transcriptomic and genomic studies also failed to uncover evidence for catalase genes [36, 37]. Despite the lack of catalase, extracts of *Perkinsus* readily removed H_2_O_2_, and were seen to possess an ascorbate-dependent peroxidase (APX) activity [22] and *P. marinus* osmotic lysates contain at least 4 mM ascorbate (Schott and Vasta, unpublished). This observation is rationalized by the finding that the *P. marinus* genome contains genes similar to those possessed by plants (*VTC1, VTC2, VTC4* of *Arabidopsis thaliana*) for synthesis of the ascorbate cofactor, in agreement with the evolution of the ascorbate biosynthesis pathway as recently described by Wheeler et al. (2015) [48]. In the present study we purified *P. marinus* APX activity, identified two genes encoding APX proteins, and characterized the phylogenetic relationships, intracellular localization and predicted 3-dimensional structures of the two encoded APX isoforms. The findings provide insight into the origins, adaptations, and function of APX in the life history of an intracellular parasite that shares evolutionary origins with both free living and parasitic protists, all of which are derived from an ancestor that likely contained an endosymbiotic alga.

Phylogenetic analysis indicates that the two APX proteins in *P. marinus* arose by gene duplication in the *Perkinsus* lineage after its divergence from dinoflagellates. A total of four ‘clades’ of APX proteins were found in the core dinoflagellates. *Oxyrrhis marina*, the non-photosynthetic dinoflagellate proposed to be basal to the dinoflagellate lineage, has APX only in clade 4, whereas the PmAPX proteins are not rooted within any one of the four dinoflagellate clades. Zámocky et al. (2014) define a group of APX-like proteins that share characteristics with cytochrome c peroxidases (CcP) [42]. Both monofunctional APX and the “hybrid A2 clade” are found in *A. carterae* (http://peroxibase.toulouse.inra.fr/). PmAPX1 and PmAPX2 are more similar to the canonical APX proteins than to the hybrid A2 clade of Zámocky et al. This is consistent with the lack of cytochrome c peroxidase activity in crude extracts of *P. marinus* (Schott and Vasta, unpublished). Protein alignments and structural models do not reveal any specific residues or features that are uniquely shared between *Perkinsus* and *Oxyrrhis*. It is also notable that the APX that is most similar to PmAPX1 and PmAPX2 is found in *Hematodinium* spp., a parasitic dinoflagellate whose life cycle alternates between a crustacean host and the environment [49, 50].

The obligately parasitic Apicomplexans (*Plasmodium, Toxoplasma*), which have numerous biochemical and structural vestiges of an ancestral plastid [51], show no genetic or biochemical evidence of APX activity [52, 53]. Therefore, according to currently understood phylogenetic relationships and their well-recognized loss or repurposing of gene families in the evolutionary process leading to adaptation of an intracellular parasitic lifestyle, the Apicomplexa must have lost APX genes, because *Vitrella brassicaformis*, within the photosynthetic sister taxon (Chromera) to Apicomplexans, does possess APX [54].

In photosynthetic eukaryotes, specific isoforms of APX are targeted to chloroplasts, mitochondria, and peroxisomes where they efficiently remove reactive oxygen species produced by light harvesting, electron transfer and metabolic activities [55]. In *P. marinus,* immunofluorescence shows that PmAPX1 is found in the cytoplasmic space, while PmAPX2, which carries a 30 aa N-terminal leader, is located at the vacuolar membrane. The PmAPX2 leader does not have transmembrane characteristics or resemble a mitochondrial import sequence, such as seen in the mitochondrially-targeted FeSOD of *P. marinus* [23]. During the purification of APX we recovered only one activity from osmotic extracts, and peptide sequence analysis detected only PmAPX1-specific (and not PmAPX2) residues. Furthermore, preliminary immunoblots of soluble proteins from digitonin-treated *P. marinus* cultures showed only one APX protein, while the cell pellet contained two immunoreactive bands (Schott and Vasta, unpublished). Together, these observations provide biochemical support that PmAPX2 is associated with only membranes. It would require additional subcellular fractionation studies and immunofluorescence to determine whether the APX activity is on the inner or outer side of the vacuolar membrane. Such studies would provide a better understanding of the signals and mechanisms for protein targeting in *P. marinus*.

By analogy to the role of APX in photosynthetic eukaryotes, the targeting of PmAPX2 to the vacuolar membrane may indicate that the organelle has high H_2_O_2_ concentrations from which the rest of the parasite cell needs to be protected. Little is known about the vacuole of *Perkinsus* spp. other than it forms early in the biogenesis of both zoospores and trophozoites and contains an electron dense “vacuoplast” whose unknown composition has been hypothesized to be an energy storage material [11]. In various taxa from bacteria to green algae, polyphosphate accumulation has been speculated to fulfill that role [56, 57]. Discovering the chemical nature of the vacuoplast would be a helpful next step in knowing whether energy storage or catabolism is occurring in the vacuole, and whether the organelle is likely to generate ROS. It is not clear whether the *Perkinsus* vacuole has a function similar to the vacuoles in related protists. In *Plasmodium* species, hemoglobin is metabolized within a vacuole, a process that generates excess ROS (16, 12, 41). In heterotrophic dinoflagellates, including *Oxyrrhis,* engulfed prey organisms are digested in the vacuole [14, 58, 59]. There are no reports on whether reactive oxygen species are produced in the degradation of prey items, and Perkins (1976) [4] pointed out that protistan food engulfment vacuoles are produced by large invaginations of the outer membrane. It would be of interest to know if *Oxyrrhis* APX is targeted to the digestive vacuole.

The three-dimensional models of APX from *P. marinus* and its taxonomic relatives reveal highly similar structures including conservation of the active site residues and architecture. The surface shape and charge of all models were also similar, with two notable exceptions: **1)** The aperture of PmAPX2 facing the delta heme edge is unique in presenting a basic surface instead of an acidic one. This could increase charge attraction for the ascorbate anion (AsA) or other negatively charged small molecules. Structural studies of APX show that AsA binds to the C6 propionate chain of the heme molecule, adjacent to the smaller gamma edge aperture [45], but in other heme peroxidases, small molecules (electron donors) also bind on the delta heme edge and may access that site through the larger proximal aperture [46]. **2)** The unique loop in the lower portion of PmAPX1 and PmAPX2 models is highly charged and extended into the solvent. The positively-charged peptide (AKSKSK) of PmAPX2 is followed by a hydrophobic motif (GLLVL). Because the modeling of the surfaces of PmAPX1 and PmAPX2 showed them to be otherwise very similar, the question arises as to whether this loop plays a role in localization of PmAPX2 at the vacuolar membrane.

The aperture facing the gamma heme edge is basic in all APX structures, but the *Oxyrrhis* aperture appears to be occluded. Even if this channel were blocked, the AsA substrate could still access the *Oxyrrhis* enzyme’s heme through the delta heme edge channel, resulting in a reduced turnover rate [46]. Given the central location of *Oxyrrhis* in the chromalveolate family, and the critical role of APX in oxidative homeostasis, it would be of great interest to see experimental confirmation of APX activity in the species, and a study of its kinetics.

Because this study of APX was motivated by the search for an activity in *P. marinus* that removes H_2_O_2_ in the absence of catalase it is worth reviewing which of the relatives of *P. marinus* contain or lack catalase (i.e., does APX “replace” catalase among the chromalveolates). Among apicomplexans, which lack APX, *Toxoplasma* (a coccidian) has catalase and *Plasmodium* (a haemosporidean) lacks it [33, 60]. Catalase is widely represented among the dinoflagellates (e.g., [61]), though there is insufficient ‘omic’ data to know if it is present in any of the parasitic dinoflagellates (syndinales), including *Hematodinium*, which possesses APX (GEMP01063346.1). Among higher plants and rhodophytes, catalase is again widespread and diverse, as is APX [62]. Among Euglenozoa, the parasites *Trypanosoma cruzi* and *Lieshmania* spp. and photosynthetic, free-living *Euglena* sp. all lack catalase and [32] possess APX. A recent paper by Kraeva et al. (2017) [63] makes the case that the loss of catalase in parasites within the Leishmaniinae is related to living within host blood and to a dixenous life history, and suggest that possesion of catalase would abrogate the low concentrations of H_2_O_2_ that are important signals to trigger parasite developmental changes. In this context, the loss of catalase by *P. marinus* would be seen not as a deficit that is compensated for by APX, but as an adaptive response to life history evolution. This is not entirely consistent, because although *P. marinus* does live in the hemal space of its oyster host, it is not a dixenous parasite.

Future empirical studies include using the APX isoform-specific antibodies developed in this study for in vivo experiments to measure the APX protein and enzyme activity levels in host-parasite interactions. Using methods developed by Alavi et al. (2009) [64], direct interactions between cultured parasites and oyster hemocytes can be studied to test how parasite recognition, engulfment, and intrahemocytic survival correlate with APX enzyme activity, and the amount and localization of the PmAPX1 and PmAPX2 proteins. These methods can even be applied to hemocytes recovered from oysters with different intensity of *P. marinus* infections.

## Conclusions

The facultative intracellular parasite *P. marinus*, which lacks catalase, is equipped to remove H_2_O_2_ with two similar APX proteins that are differentially targeted to the cytoplasm and vacuolar membrane. The corresponding genes encoding for these proteins, typically present in photosynthetic organisms from microalgae to higher plants, appear to have arisen by gene duplication after the divergence of the Perkinsozoa and the Dinozoa. Structural predictions indicate that the two isoforms have different surface charges and active site accessibility by the substrate. These discoveries raise questions about the specific or differential roles of PmAPX1 and PmAPX2 in removing H_2_O_2_ generated by cellular metabolism and by exogenous sources such as a host innate immune response. Although phylogenetic analyses based on single or multiple genes have placed the Perkinsozoa as a key taxon between the apicomplexan parasites and the photosynthetic dinoflagellates [5, 37], this study contributes new biochemical, molecular, and structural evidence to support the photosynthetic ancestry of the Perkinsozoa from a functional standpoint.

## Methods

### Parasite culture

*Perkinsus marinus* strain Texas (ATCC 50849) was cultured in 1 part DMEM:2 parts Ham’s F12 (Sigma, St. Louis, MO) with 5% heat inactivated fetal bovine serum (Paragon, Atlanta GA), containing 15 ppt artificial sea salt (Crystal Sea, Marinemix, Baltimore, MD) as previously described [23]. Routine propagation was conducted without agitation at 28 °C in flat-bottom tissue culture flasks. Propagation for APX purification was conducted in 1 l Erlenmeyer flasks shaking at 100 rpm.

### Molecular techniques

Restriction enzymes and other enzymes for manipulation of DNA were purchased from Promega (Madison, WI). Taq polymerase, buffers and dNTPs were purchased from Takara Scientific (Takara Mirus Bio Inc., Otsu, Shiga, Japan). Dye terminator sequencing (ABI PRISM® BigDye™ Primer Cycle Sequencing Kit, Foster City, CA) was conducted by thermal cycle reactions (PTC-200 cycler, MJ Research, Inc., Watertown, MA) and resolved on an Applied Biosystems 373 sequencer (Foster City, CA). Degenerate and inosine-containing oligonucleotide primers were synthesized by Invitrogen Inc. (Carlsbad, CA, USA). Plasmid vector-targeted oligonucleotides (T7, T3; MCS1, MCS2) used for PCR-based confirmation of plasmid constructions were synthesized by the Bioanalytical Services Lab at the University of Maryland Center of Marine Biotechnology. Plasmids were propagated in *E. coli* strains JM109 and DH5α grown in LB agar at 37 °C with 100 μg ml^− 1^ ampicillin.

### Ascorbate peroxidase assays

Ascorbate peroxidase activity (EC1.11.1.11) was measured spectrophotometrically (Shimadzu model UV-1601) by monitoring the H_2_O_2_-dependent oxidation of ascorbate at 290 nm, based on the procedure of Nakano and Asada (44). Assay buffer (50 mM NaPO_4_, pH 7.2, 0.4 mM ascorbate), 0.2 ml, was placed in a quartz cuvette, followed by enzyme solution (1–5 μl) and the A_290_ monitored for 20 s. Ten μl of 10 mM H_2_O_2_ was added, and the decrease in A_290_ monitored for 40 s. APX activity was calculated from the A_290_ slope for 20 s following hydrogen peroxide addition. The coefficient (A290) used for calculating ascorbate concentration was 2.8 mM^− 1^ cm^− 1^. In-gel visualization for APX activity of proteins separated by nondenaturing PAGE, was conducted according to the nitroblue tetrazolium procedure of Mittler and Zalinskas [39].

### Purification of APX

The *P. marinus* APX purification strategy was based on published methods for plant APX activity (44). Late log phase trophozoites were harvested by centrifugation (500 x g 5 min), and the cell pellet (5 g) was washed in 500 mM NaCl with 25 mM NaPO_4_, pH 7.3. A hypotonic lysate of *P. marinus* trophozoites was prepared by rapidly diluting the cell pellet (at 0 °C) in 10 volumes of 25 mM NaPO_4_, pH 7.3, 0.5 mM phenylmethylsulfonyl fluoride, 0.5 mM EDTA, and 2 mM ascorbate, and vortexing vigorously for 30 s. The extract was clarified by centrifugation for 10 min at 8000 x g at 4 °C, and the supernatant passed through a 0.45 μm filter. Clarified hypotonic extract was diluted 2-fold with 25 mM NaPO_4_, pH 7.4, and loaded (16.1 mg protein) onto a DEAE-Sephacel column equilibrated with 25 mM NaPO_4_, pH 7.4. The column was eluted with a step gradient of NaCl and fractions monitored for APX activity. Peak activity, in the 250–300 mM steps, was pooled and concentrated (Centricon 10, Millipore) for fractionation by hydrophobic interaction chromatography. Peak activity DEAE (2.8 mg protein) was brought to 1.5 M (37%) (NH_4_)_2_SO_4_, clarified by centrifugation for 10 min at 10,000 rpm (20 °C) and loaded onto a 1.5 ml phenyl sepharose column (Pharmacia) equilibrated with 35% (NH_4_)_2_SO_4_. Elution was by step gradient with decreasing (NH4)_2_SO_4_ concentration from 50 to 0%. Peak APX activity eluted in the 28% (NH_4_)_2_SO_4_ step. The peak activity fraction, as well as the fraction before and after, were pooled (33 μg protein) in a total of 1.5 ml and concentrated to 0.1 ml.

### Peptide sequencing of APX

Peak fractions from the phenyl Sepharose stage of the second purification were pooled, desalted by dialysis against 25 mM NaPO_4_, and separated by 10% SDS-PAGE. After electrophoresis, the gel was stained with Coomassie blue to visualize major protein bands. The putative PmAPX protein band (32 kDa) was excised and analyzed by tryptic digestion, HPLC, and mass spectrometry (MS/MS) at the Microchemical Facility at Emory University. Resulting peptides were separated by capillary RP-HPLC and the fractions absorbing at 210 nm were collected and sequenced using Edman degradation. The mass of the peptides agreed with their mass calculated based on the sequences determined; the MS/MS spectra were also in agreement with these sequences.

### Amplification of APX genes, and isolation of full length cDNA and gDNA

Based on experimentally-determined peptide sequences, the following degenerate oligonucleotides were designed and synthesized: Peptide A: APXf6, TTYGCYTGGCAYGAYWSIGGIAC; Peptide B: APXr7, CIGGIGGRCAYTCYTCIGGICC.

Amplifications were conducted using a touchdown strategy on *P. marinus* cDNA and gDNA libraries, constructed in lambda Zap and lambda DASH (Stratagene), previously described [20, 26], using Taq polymerase (ExTaq, Takara Mirus Bio). The ~ 300 bp PCR product was sequenced and determined by BLAST analysis (not shown) to be similar to known APX genes. From this sequence, additional primers were designed using the program Primer3 [27]. Primers APXR8 (CCCCCACAGTGTTTAAGG) and APXF9 (AGCGGCGGCATCATCTAT) were used in conjunction with library vector primers MCS1 (3′-GCCGCTCTAGAACTAGTGGAT-5′), MCS2 (3′-TATAGGGCGAATTGGGTACCG-5′), T3 (3′-ATTAACCCTCACTAAAG-5′) and T7 (3′-AATACGACTCACTATAG-5′) to amplify the 5′ and 3′ ends of putative APX genes from the aforementioned cDNA and gDNA libraries. Sequences of amplicons generated by APXF9 and T7 from both genomic and cDNA templates showed heterogeneity at numerous nucleotide locations, suggesting that there were two APX-like genes in the cDNA and genomic libraries. To discriminate between putative *PmAPX1* and *PmAPX2* genes, amplicons extending from vector primers to the internal primers APXF9 and APXR8 were cloned in the Topo TA vector (Invitrogen) and sequenced. Consensus PmAPX1 and PmAPX2 sequences were assembled using Sequencher (Gene Codes Corp., Ann Arbor, MI, USA).

### Phylogenetic analyses

For phylogenetic analysis potential APX sequences based on functional analysis described above were used as BLAST queries against a suite of seven core dinoflagellates and *Oxyrrhis marina*. The results from these searches were then used iteratively and added to the query file to ensure that no additional putative APX were found. Putative APX from *P. marinus* and *Amphidinium carterae* were used as queries in a conserved domain search against the NCBI conserved domain databases. For outgroups APX text queries to tobacco *Nicotiana tabacum* protein sequences were used as a seed for further queries against diatoms, cryptophytes, other green plants, apicomplexans, chromerids, and trypanosomes. Sequences were aligned using MUSCLE [65] and the alignment inspected by eye to ensure that conserved sites were aligned. Phylogenetic analyses were performed with RAxML and the PROTGAMMAJTT model with 100 rapid bootstrap replicates [66]. The phylogeny was performed with two major iterations, to place the *P. marinus* and core dinoflagellate sequences into the context of a very broad set of APX including trypanosomes and cytosolic versions from plants. Following this analysis a second alignment was constructed after trimming the cytosolic versions from plants, diatoms and trypanosomes. The alignment was rerun using MUSCLE, followed by manual inspection and used as input for RAxML as described above.

### Antipeptide antibodies against PmAPX1 and PmAPX2

Specific antisera were raised in rabbits against synthetic peptides corresponding to the PmAPX1-specific region (VRFDAELKHEANAGLAKGRR, pI of 9.98) and the PmAPX2-specific region (IIYDVELSDAANAGLPKALK, pI of 4.56), highlighted in Fig. 3a. A pan-APX antisera was also raised against peptide B (DISGPEECPPEGR) (Fig. 2d) that is 93% identical between PmAPX1 and PmAPX2. A “pan-APX” antibody was raised against the peptide DISGPEECPPEGRL of PmAPX1, which is nearly identical to the peptide DISGPEECPPAGRL of PmAPX2. Each peptide was synthesized, conjugated to KLH, and injected into a total of 6 rabbits according to a standard 70 day protocol. Rabbit immunizations, bleeding and euthanasia were performed by a company (Open Biosystems Inc./ThermoFisher) accredited by the United States Department of Agriculture (USDA) and Office of Laboratory Animal Welfare (OLAW). IgG was isolated from antiserum by protein A-Sepharose chromatography, and specific antibodies were isolated by affinity purification on recombinant PmAPX1 and PmAPX2 immobilized on CNBr activated sepharose 4B (Sigma). Antibody titers were assessed by ELISA, using the synthesized APX peptides described above. Specificity of each antibody was verified on blots of recombinant PmAPX1 and PmAPX2 (data not shown) and extracts of *P. marinus* trophozoites (Additional file 3: Figure S2).

### Production of recombinant PmAPX1 and PmAPX2

PmAPX1 and PmAPX2 were engineered for expression with N-terminal 6xHIS and S-tag affinity domains from the pET30-EK-LIC vector (Novagen) by amplification of complete coding regions of PmAPX1 and PmAPX2 from the aforementioned cDNA library (20), using primers that incorporated Ligation Independent Cloning compatible ends. Forward and reverse PmAPX1 primers (LIC vector sequences underlined) were 5′-GACGACGACAAG-ATGGTACAGAGTGATCCCAAGC-3′ and 5′-GAGGAGAAGCCCGG-TTTATCACATTTTGATGCCACCATCTAC-3′, respectively. Forward and reverse primers for PmAPX2 were 5′-GACGACGACAAG-ATGGGAAACATCTGTGACTCC-3′, and 5′-GAGGAGAAGCCCGG-TTTATCATACCTTGATCCCACCGTC-3′, respectively. Proteins were expressed in *E. coli* BL21(DE3), using IPTG induction for 2–4 h at 30 °C. Recombinant protein was recovered by sonication of bacterial pellets at 0 °C in 300 mM NaCl, 50 mM NaPO_4_, pH 7.0, with 0.5 mM PMSF. Extracts were clarified by centrifugation at 8000 x g at 4 °C, and recombinant proteins enriched by immobilized metal affinity chromatography on Talon resin (BD Biosciences Clontech, San Jose, CA, USA) according to the manufacturer’s protocol.

### Genomic DNA blots

Total genomic DNA from *P. marinus* strain Texas (ATCC 50983), prepared by proteinase K digestion and phenol/chloroform extraction (32) was digested for 18 h at 37 °C with restriction enzymes in appropriate buffers. Digested DNA was separated on 1% agarose gels, transferred to Hybond N membrane (Amersham) by alkaline transfer, and fixed by baking under vacuum. Blots were hybridized with PCR amplicons corresponding to specific fragments of PmAPX1 and PmAPX2, labeled by random priming (Redi-prime, Amersham) to incorporate deoxycytidine 5-triphosphate -^32^P (Amersham). Hybridizations were conducted for 16 h at 63 °C, and blots were washed in 0.2X SSC, 0.1% SDS, at 50 °C. PCR primers used to amplify the PmAPX1 probe were APXf14: 5′-TTGTGCCTCGCTATGTTAATG-3′, and APXr17: 5′-GTCTTTGGCGTATTGCTTGG-3′. PmAPX2 was amplified with APXf12: 5′-CGAGTCTTGGCTCAAGTTTG-3′, and APXr16: 5′-TGATGGGATCACAGTTCAGC-3′. Autoradiography was conducted with an intensifying screen at − 80 °C for 12–48 h, using Kodak X-ray film.

### Immunoblots

Soluble protein extracts of *P. marinus* trophozoites were prepared by lysis of freshly cultured trophozoites with 25 mM NaPO_4_ buffer containing 0.5% TX-100, 0.5 mM PMSF, and 0.5 mM EDTA. Protein was electrophoresed in SDS 10% PAGE gels and electrophoretically transferred to polyvinylidene difluoride (PVDF) membrane in 25 mm Tris, 190 mm glycine, and 10% (v/v) methanol, at 40 V for 60 min, at 4 °C. PVDF membranes were blocked in 1% nonfat dry milk/PBS for 30 min at room temperature prior to addition of primary antibody in PBS. After 12–16 h in primary antibody (4 °C), blots were washed 4X with PBS + 0.05% Tween 20, then incubated in HRP conjugated goat-anti rabbit IgG (Bio-Rad) for 1 h at room temperature. After four washes in PBS-Tween 20, blots were developed with the chemiluminescent substrate luminol (Pico-west kit, Pierce), and light production was recorded by exposure to x-ray film (Kodak).

### Subcellular localization of APX by immunofluorescence and confocal microscopy

*P. marinus* trophozoites were fixed with 3% formaldehyde (Fisher, preserved 37% reagent) for 15 min, followed by addition of Triton X-100 to 0.1% for another 15 min at room temperature. After 3 washes in PBS, fixed cells were incubated for 10 min in 3% normal goat serum, followed by addition of primary antibody at 1:600, and incubated at 4 °C overnight. Following 3 washes in PBS, cells were incubated with (1:500) goat-anti-mouse-Alexa 488 (Molecular Probes/ThermoFisher) for 1 h at room temperature. After 3 washes in PBS, cells were mounted on polylysine coated slides, observed and photographed on an Olympus fluorescence microscope. For confocal imaging of antibody-stained cells, fixed and antibody-stained *P. marinus* trophozoites from an actively growing culture were incubated on polylysine costed slides as described for immunofluorescence. Optical sections were imaged at 63x on a Zeiss Pascal 5 Confocal Scanning Laser Microscope using an argon laser.

### Homology modeling

Three-dimensional models were generated using the SWISS-MODEL server (https://spdbv.vital-it.ch [67]), using as a template the crystal structure of the chloroplastic APX of tobacco (PDB ID 1iyn), [40]. Models based on initial sequence alignments were examined to correct the alignments to ensure that insertions and/or deletions did not occur in regions of secondary structure. The sequence identities between targets and template ranged from 54 to 62%. The quality of the final models was assessed using the criteria provided by the modeling platform and by running the structure evaluation program MolProbity 4.4 [68].

### Graphical presentation and statistical analysis

Figure 1 was generated with Kaleidagraph 4.5. Figure 3b was created using the protein translations of accession numbers XM_002767244 and XM_002767239 with Swiss PDBViewer 3.9b, using tobacco APX as a guide (PDB ID 1iyn). Three-dimensional models (Figures 7 and 8, Additional file 5: Figure S4) were constructed on the SWISS-MODEL server (https://spdbv.vital-it.ch) [67]. Phylograms (Figures 4 and 5) were created using with RAxML with alignments created in MUSCLE.

## Additional files

Additional file 1: **Figure S1** Autoradiograms of genomic DNA blots. Duplicate blots of *P. marinus* genomic DNA digested with EcoRI, HindIII, SalI, XbaI, and XhoI were probed with 32-P labeled oligonucleotides specific to *PmAPX1* (left) and *PmAPX2* (right). (DOCX 162 kb)

Additional file 2: **Table S1** Accession numbers for taxa phylogenetic analysis depicted in Fig. 5. GenBank accession numbers for all of the protein sequences used in phylogenetic analyses. (DOCX 83 kb)

Additional file 3: **Figure S2** Demonstration of antibody specificity. Specific anti-APX1 and anti-APX2 rabbit IgG, raised against peptides RFDAELKHEANAGLAKGRR and IYDVELSDAANAGLP, respectively, were used to probe immunoblots of total whole cell extracts and label fixed trophozoites of *P. marinus*. *A,* anti-PmAPX1, raised against RFDAELKHEANAGLAKGRR. *B*, anti-PmAPX2, raised against IYDVELSDAANAGLP. *C*, anti-PmAPX1/PmAPX2, raised against DISGPEECPPEGRL. *D*, the anti-PmAPX1/2 antiserum was used to probe a blot of pellet and supernatant fractions of digitonin-extracted and *P. marinus* proteins. (DOCX 260 kb)

Additional file 4: **Figure S3** Alignment of APXs from selected taxa. Representative APXs from the major branches in the phylogeny depicted in the inset of Fig. 4b (*Perkinsus*, *Oxyrrhis*, Syndineans, core dinoflagellates, chromerids, and apicomplexans) were aligned with plastidic and cytosolic APX, including soybean APX for which there is a 3D structure. The N-terminal leaders of plastidic forms have been trimmed away. Key active site residues are conserved among all APXs and are highlighted on the alignment. This includes Trp41, His42, Arg38 above the heme molecule, Ser160, His163, Trp179, Asp208 and below the heme, and His169, Arg172 coordinating the substrate. Two insertions that are characteristic of plastid isoforms are visible starting at *Nicotiana* APX residue Cys133 and Gly177. (DOCX 730 kb)

Additional file 5: **Figure S4** Ribbon overlay of PmAPX1 and PmAPX2. PmAPX1 in yellow; PmAPX2 in salmon. *A,* Overall ribbon overlay of PmAPX1 and PmAPX2 showing the position of the loop with the KSKSK peptide of PmAPX2 in magenta. *B,* Main chain differences in the KSKSK loop. *C,* Residues of the KSKSK loop of APX2 (atoms are represented by spheres; C in magenta; N in blue; O in red). (DOCX 309 kb)

## Data Availability

The nucleotide and amino acid sequences generated and/or analyzed during the current study are all available as the accession numbers listed in the text within GenBank [https://www.ncbi.nlm.nih.gov/genbank/]. Alignments of putative APX proteins from core dinoflagellates and *P. marinus* are available as Study 23146 at https://treebase.org(http://purl.org/phylo/treebase/phylows/study/TB2:S23146?x-accesscode=858c9a69dc0247feeb40006d0b6b8a0a&format=html).

## Abbreviations

APX: Ascorbate peroxidase
ASC: Ascorbic acid
MDA: Monodehydroascorbate
MPO: Myeloperoxidase
PAGE: Polyacrylamide gel electrophoresis
ROS: Reactive oxygen species
SDS: Sodium dodecyl sulfate
SOD: Superoxide dismutase
