# Supplementary material for: Lacking catalase, a protistan parasite draws on its photosynthetic ancestry to complete an antioxidant repertoire with ascorbate peroxidase
Source: BMC Evol Biol. 2019 Jul 19;19:146. doi: 10.1186/s12862-019-1465-5 (PMC6642578; doi:10.1186/s12862-019-1465-5)
Supplement: Supplementary file 1 — Figure S1 Autoradiograms of genomic DNA blots. Duplicate blots of P. marinus genomic DNA digested with EcoRI, HindIII, SalI, XbaI, and XhoI were probed with 32-P labeled oligonucleotides specific to PmAPX1 (left) and PmAPX2 (right). (DOCX 162 kb) [file 12862_2019_1465_MOESM1_ESM.docx]

Fig S1. Autoradiograms of genomic DNA blots


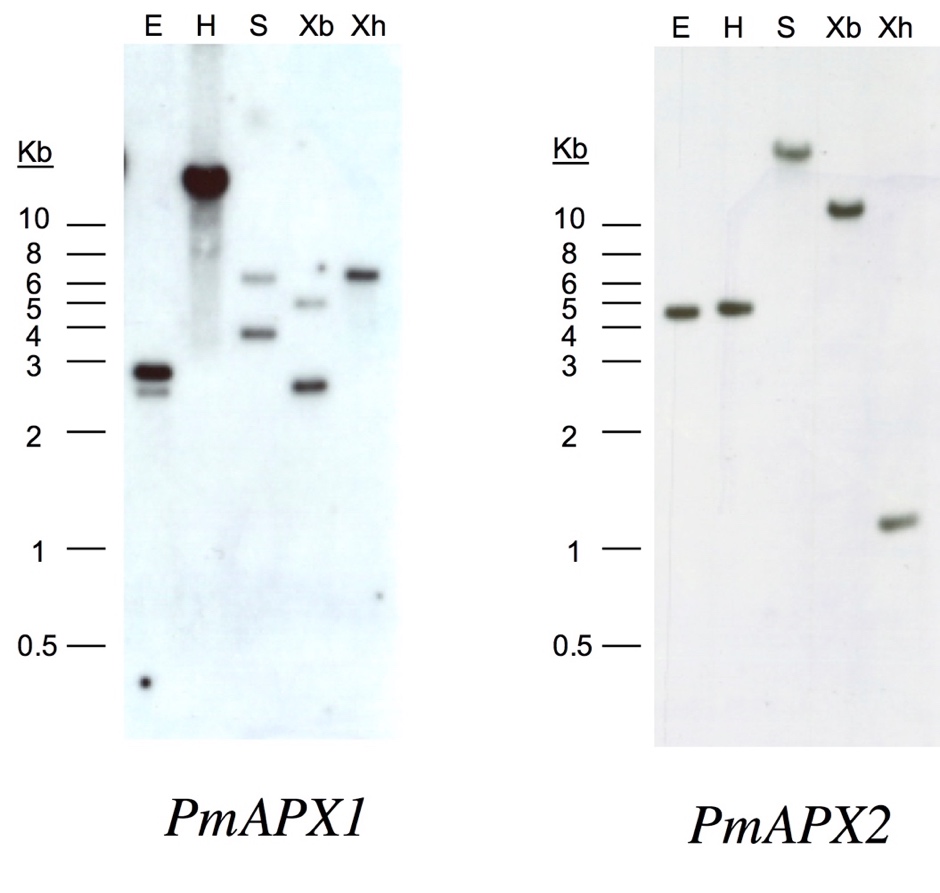


**Fig**. **S1. Autoradiograms of genomic DNA blots**. Duplicate blots of *P. marinus* genomic DNA digested with EcoRI, HindIII, SalI, XbaI, and XhoI were probed with 32-P labeled oligonucleotides specific to *PmAPX1* (left) and *PmAPX2* (right).
