# Supplementary material for: Lacking catalase, a protistan parasite draws on its photosynthetic ancestry to complete an antioxidant repertoire with ascorbate peroxidase
Source: BMC Evol Biol. 2019 Jul 19;19:146. doi: 10.1186/s12862-019-1465-5 (PMC6642578; doi:10.1186/s12862-019-1465-5)
Supplement: Supplementary file 2 — Table S1 Accession numbers for taxa phylogenetic analysis depicted in Fig. 5. GenBank accession numbers for all of the protein sequences used in phylogenetic analyses. (DOCX 83 kb) [file 12862_2019_1465_MOESM2_ESM.docx]

**Table S1 Accession numbers for taxa phylogenetic analysis depicted in Fig. 5.** GenBank accession numbers for all of the protein sequences used in phylogenetic analyses.

| **Species name** | **Accession** |
| --- | --- |
| Dinoflagellates |  |
| *Scrippsiella trochoidea* | SRX551166-8 |
| *Karlodinium veneficum* | SRX730948, SRX730949 |
| *Symbiodinium* B1 | SRX021274, SRX021275 |
| *Amphidinium carterae* | SRX722011 |
| *Karenia brevis* | SRX551359, SRX551258-61 |
| *Akashiwo sanguineum* | SRX730945, SRX730946 |
| *Kryptoperidinium foliaceum* | SRX551248-9 |
| *Crypthecodinium cohnii* | SRX551367-8, SRX551296-7 |
| *Hematodinium* sp*.* | GEMP01063346 |
| *Oxyrrhis marina* | ACE81819, SRX554277-9 |
|  |  |
| *Vitrella brassicaformis* | CEM12648.1 multiple isoforms, CEM29166 |
| *Guillardia theta* | XP_005828604.1 |
|  |  |
| Diatoms |  |
| *Phaeodactylum tricornutum* CCAP_1055 | XP_002181742 |
| *Thalassiosira pseudonana* CCMP1335 | XP_002291359 |
| *Phaeodactylum tricornutum* CCAP_1055 | XP_002181744 |
|  |  |
| Green plants |  |
| *Brachypodium distachyon* | XP_003578843 |
| *Setaria itailica* | XP_004977222 |
| *Jatropha curcas* | XP_012078303.1 |
| *Populus euphratica* | XP_011027505 |
| *Nicotiana tabacum* APX6 chloroplastic isoformX2 | XP_016437245 |
| *Nicotiana tabacum* APX6 chloroplastic isoformX1 | XP_016469841 |
| *Nicotiana tabacum* stromal | BAA78553, pdb\|1IYN_BAC10691 |
| *Nicotiana tabacum* thylakoid bound | BAA78552 |
| *Medicago truncatula* | XP_003602044 |
| *Amborella trichopoda* | XP_006847188 |
| *Elaeis guineensis* | XP_010930723 |
| *Phoenix dactylifera* | XP_017696878 |
