# Supplementary material for: Lacking catalase, a protistan parasite draws on its photosynthetic ancestry to complete an antioxidant repertoire with ascorbate peroxidase
Source: BMC Evol Biol. 2019 Jul 19;19:146. doi: 10.1186/s12862-019-1465-5 (PMC6642578; doi:10.1186/s12862-019-1465-5)
Supplement: Supplementary file 3 — Figure S2 Demonstration of antibody specificity. Specific anti-APX1 and anti-APX2 rabbit IgG, raised against peptides RFDAELKHEANAGLAKGRR and IYDVELSDAANAGLP, respectively, were used to probe immunoblots of total whole cell extracts and label fixed trophozoites of P. marinus. A, anti-PmAPX1, raised against RFDAELKHEANAGLAKGRR. B, anti-PmAPX2, raised against IYDVELSDAANAGLP. C, anti-PmAPX1/PmAPX2, raised against DISGPEECPPEGRL. D, the anti-PmAPX1/2 antiserum was used to probe a blot of pellet and supernatant fractions of digitonin-extracted and P. marinus proteins. (DOCX 260 kb) [file 12862_2019_1465_MOESM3_ESM.docx]

Fig S2. Demonstration of antibody specificity


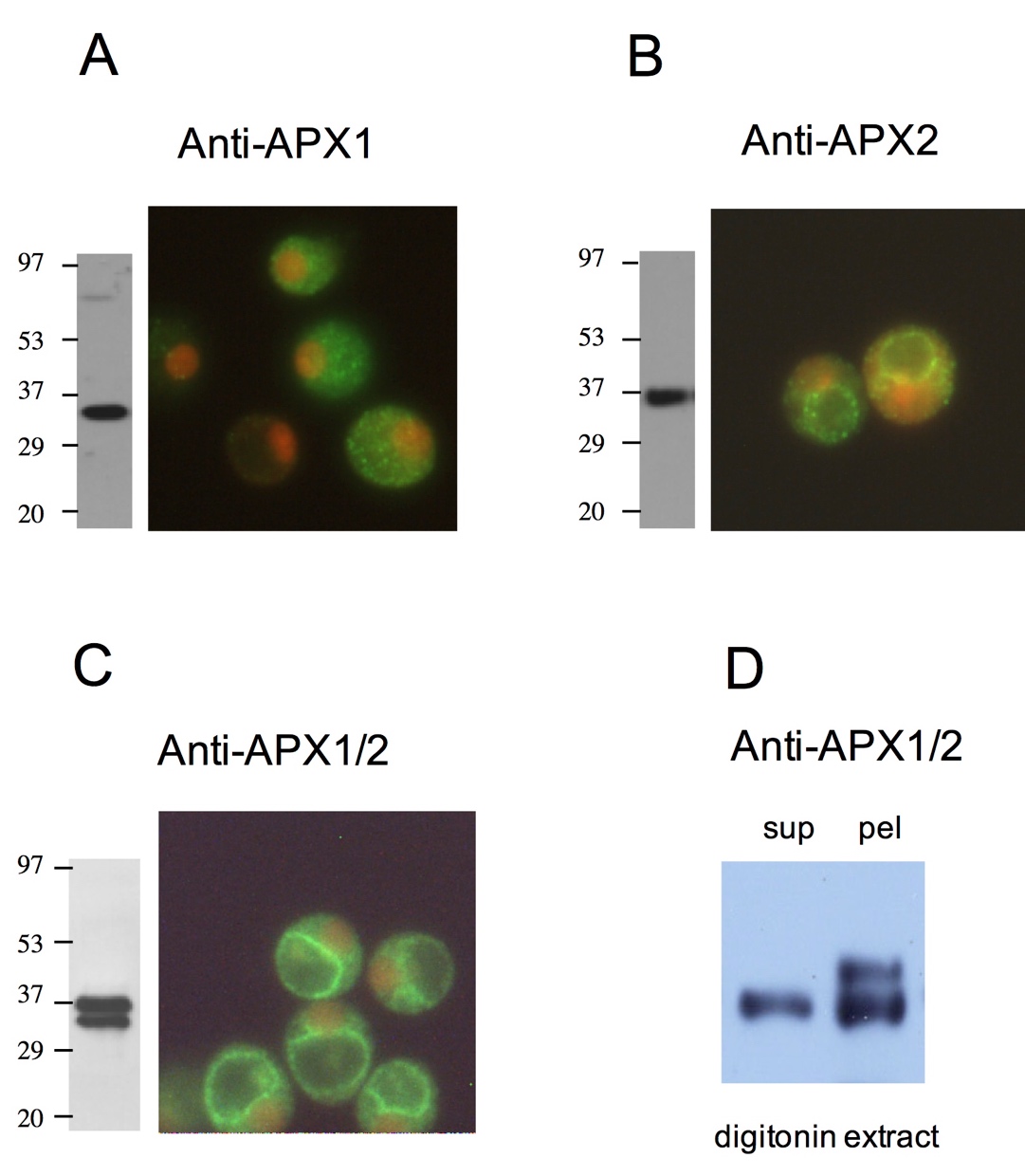


**Fig. S2. Demonstration of antibody specificity**. Specific anti-APX1 and anti-APX2 rabbit IgG, raised against peptides RFDAELKHEANAGLAKGRR and IYDVELSDAANAGLP, respectively, were used to probe immunoblots of total whole cell extracts and label fixed trophozoites of *P. marinus*. *A,* anti-PmAPX1, raised against RFDAELKHEANAGLAKGRR. *B*, anti-PmAPX2, raised against IYDVELSDAANAGLP. *C*, anti-PmAPX1/PmAPX2, raised against DISGPEECPPEGRL. *D*, the anti-PmAPX1/2 antiserum was used to probe a blot of pellet and supernatant fractions of digitonin-extracted and *P. marinus* proteins.
