# Supplementary material for: Lacking catalase, a protistan parasite draws on its photosynthetic ancestry to complete an antioxidant repertoire with ascorbate peroxidase
Source: BMC Evol Biol. 2019 Jul 19;19:146. doi: 10.1186/s12862-019-1465-5 (PMC6642578; doi:10.1186/s12862-019-1465-5)
Supplement: Supplementary file 4 — Figure S3 Alignment of APXs from selected taxa. Representative APXs from the major branches in the phylogeny depicted in the inset of Fig. 4b (Perkinsus, Oxyrrhis, Syndineans, core dinoflagellates, chromerids, and apicomplexans) were aligned with plastidic and cytosolic APX, including soybean APX for which there is a 3D structure. The N-terminal leaders of plastidic forms have been trimmed away. Key active site residues are conserved among all APXs and are highlighted on the alignment. This includes Trp41, His42, Arg38 above the heme molecule, Ser160, His163, Trp179, Asp208 and below the heme, and His169, Arg172 coordinating the substrate. Two insertions that are characteristic of plastid isoforms are visible starting at Nicotiana APX residue Cys133 and Gly177. (DOCX 730 kb) [file 12862_2019_1465_MOESM4_ESM.docx]

Fig S3. Alignment of APXs from selected taxa


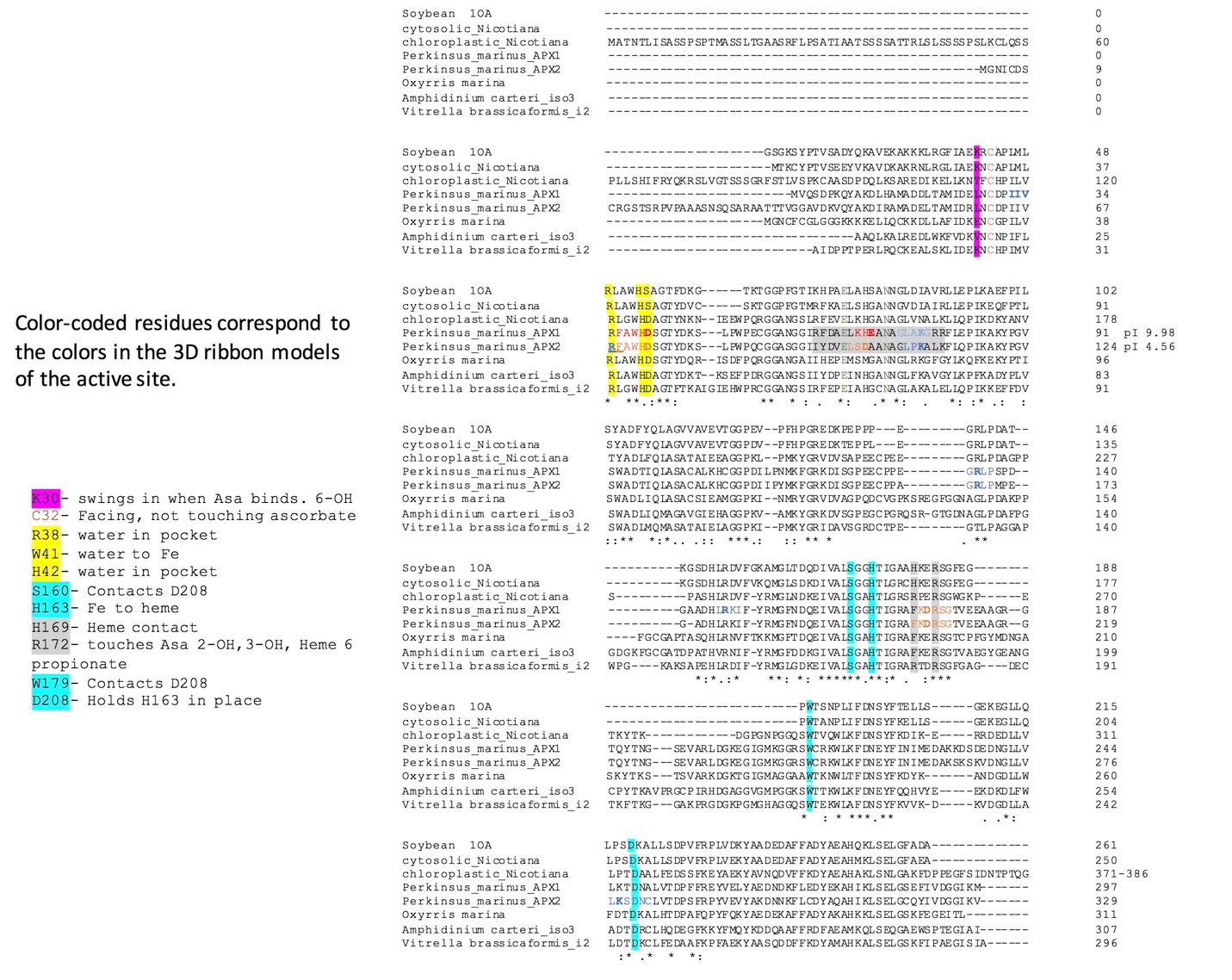


**Fig**. **S3. Alignment of APXs from selected taxa**. Representative APXs from the major branches in the phylogeny depicted in the inset of **Fig. 4B** (*Perkinsus*, *Oxyrrhis*, Syndineans, core dinoflagellates, chromerids, and apicomplexans) were aligned with plastidic and cytosolic APX, including soybean APX for which there is a 3D structure. The N-terminal leaders of plastidic forms have been trimmed away. Key active site residues are conserved among all APXs and are highlighted on the alignment. This includes Trp41, His42, Arg38 above the heme molecule, Ser160, His163, Trp179, Asp208 and below the heme, and His169, Arg172 coordinating the substrate. Two insertions that are characteristic of plastid isoforms are visible starting at *Nicotiana* APX residue Cys133 and Gly177.
