# Supplementary material for: Lacking catalase, a protistan parasite draws on its photosynthetic ancestry to complete an antioxidant repertoire with ascorbate peroxidase
Source: BMC Evol Biol. 2019 Jul 19;19:146. doi: 10.1186/s12862-019-1465-5 (PMC6642578; doi:10.1186/s12862-019-1465-5)
Supplement: Supplementary file 5 — Figure S4 Ribbon overlay of PmAPX1 and PmAPX2. PmAPX1 in yellow; PmAPX2 in salmon. A, Overall ribbon overlay of PmAPX1 and PmAPX2 showing the position of the loop with the KSKSK peptide of PmAPX2 in magenta. B, Main chain differences in the KSKSK loop. C, Residues of the KSKSK loop of APX2 (atoms are represented by spheres; C in magenta; N in blue; O in red). (DOCX 309 kb) [file 12862_2019_1465_MOESM5_ESM.docx]

Fig S4. Ribbon overlay of PmAPX1 and PmAPX2


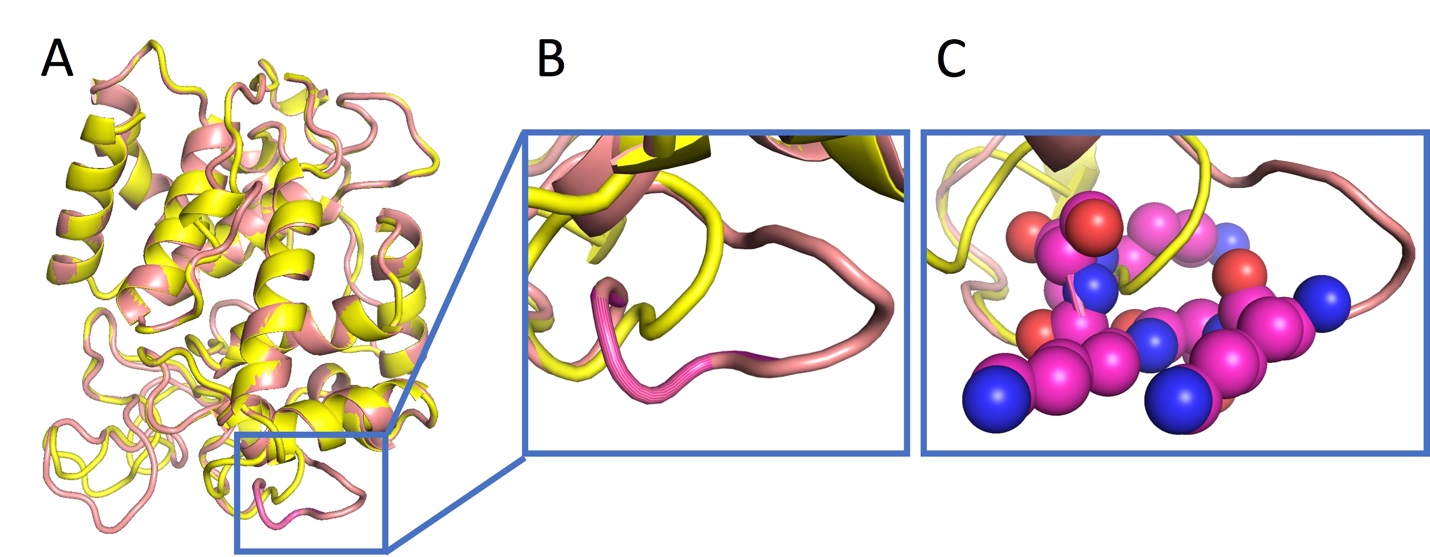


**Fig. S4. Ribbon overlay of PmAPX1 and PmAPX2**. PmAPX1 in yellow; PmAPX2 in salmon. *A,* Overall ribbon overlay of PmAPX1 and PmAPX2 showing the position of the loop with the KSKSK peptide of PmAPX2 in magenta. *B,* Main chain differences in the KSKSK loop. *C,* Residues of the KSKSK loop of APX2 (atoms are represented by spheres; C in magenta; N in blue; O in red).
